# A Seventeen-Year Descriptive Study of Infective Endocarditis Features at a Tertiary, Teaching Hospital

**DOI:** 10.7759/cureus.15983

**Published:** 2021-06-28

**Authors:** Saleh A Alghamdi, Abdulaziz M Alkhammash, Abdulrahman F Alotaibi, Saeed A Bugshan, Nawaf K Alshanbri, Mohammed Zahrani

**Affiliations:** 1 College of Medicine, King Abdulaziz University, Jeddah, SAU; 2 Internal Medicine, King Abdulaziz University, Jeddah, SAU

**Keywords:** infective endocarditis, echocardiography, vegetation, modified duke criteria, sepsis, embolization

## Abstract

Introduction

Infective endocarditis (IE) is a microbial infection affecting the endothelial lining of the heart chambers and valves. Despite advances in diagnostic methods and management, IE still carries high levels of morbidity and mortality. There are no comprehensive data describing incidence, risk factors, and outcomes of IE in Saudi Arabia.

Our study aims to describe the epidemiological and clinical characteristics and outcomes of adult patients with IE treated in a tertiary, Teaching Hospital.

Methods

This is a descriptive, retrospective review of charts (between January 2003 and June 2019) conducted at King Abdulaziz University Hospital. We included all patients 16 years and older with a definitive diagnosis of IE based on Duke Criteria. We collected the following information: demographics, risk factors, comorbidities, microbial type, antibiotic choice, complications, laboratory data, echocardiography data, and mortality.

Results

We found a total of 60 adult patients with definitive diagnoses of IE: 55% of them were males, and the mean age was 48.71 ± 18.3 years. Hypertension was the most common comorbidity, affecting 23 patients (38.3%), followed by end-stage renal disease (ESRD) in 20 patients (33.3%) and diabetes in 17 (28.3%). *Staphylococcus aureus* was the most common organism (25%), and vancomycin was the most prescribed antibiotic. Fifty-eight patients were treated medically (96.5%). Furthermore, 88% of the patients had vegetations, detected by echocardiography, and the majority had single-valve involvement. Sepsis was the most common complication, and the mortality rate was 36.7%.

Conclusion

There was a small number of definitive IE cases over a 17-years span in our institution. Surprisingly, a higher mortality rate was found among our patients and a lower surgical intervention rate in comparison with the literature. Though we acknowledge the limitations of a retrospective, chart review study, we can speculate that the high mortality rate may be in part related to a higher number of virulent organisms, delayed presentation, and lack of prompt surgical intervention.

## Introduction

Infective endocarditis (IE) is a life-threatening, multisystemic illness initiated by microbial infection, mostly bacterial, targeting the endothelial lining of the heart chambers and valves. It could also affect the mural endothelium, chordae tendineae, interventricular septum, and other intracardiac tissues or devices [1-3].

The modified Duke Criteria is the recommended diagnostic method to follow for diagnosing IE according to recent guidelines [4]. However, despite the huge advances in diagnostic methods as well as antimicrobial, medical, and surgical techniques of management nowadays, IE still shows grave levels of morbidity and mortality [5,6], with a five-year mortality rate exceeding 40% [7]. IE could also result in lethal conditions such as embolic events (most seriously in the form of embolic stroke), disseminated infections throughout the body, congestive heart failure, and organ failure; and these complications are common. In addition, IE could deteriorate to DIC or lead to fatal multiorgan failure [8]. The incidence of at least one complication could affect as much as half the patients diagnosed with IE [9].

Clinical risk factors for IE include the following: advanced age (above 58 years), male gender, patients who have prolonged vascular access, intravenous drug use, implanted intracardiac devices, as well as prosthetic valves. Furthermore, several comorbidities have been correlated with increased risk of IE development, including chronic kidney disease (CKD), congenital heart diseases (CHD), cancer, human immunodeficiency virus (HIV), diabetes mellitus (DM), and previous dental procedures. Furthermore, contact with the healthcare system itself is considered a risk factor for developing IE [7,10-13].

An important predisposing factor is a chronic hemodialysis (CH). Patients with CKD are more susceptible to infections and the mortality rate is high, where half of these patients will not survive [14,15]. CH patients have a higher incidence of IE compared with those receiving peritoneal dialysis or kidney transplantation [16].

In developing countries, IE continues to be related to rheumatic heart disease (RHD), while in developed countries, patients are older and referred to healthcare facilities, and degenerative or prosthetic valves, as well as cardiovascular implantable electronic devices, have replaced RHD as the trending risk factor [17,18]. A study has concluded that patients with simple congenital ventricular septal defects (VSDs) have up to a 15‐fold increased risk of IE compared with the general population, and those with unrepaired VSDs have a considerably higher risk [19].

Globally, the incidence of IE ranges from 1.5 to 11.6 cases per 100,000 persons per year, peaking at 15 patients per 100,000 of the population in the United States (US) in 2011. The male to female ratio is over 1.5:1 [1,2,20,21]. The most commonly affected valve in IE patients is the mitral valve, followed by the aortic, tricuspid valve, and less frequently, the pulmonary valve [22].

According to a recent huge prospective study done in Turkey, *Staphylococcus aureus* is the most common causative organism behind IE, which has been found in more than a third of patients, followed by *Streptococcus viridans*, and Enterococci [23]. Another 10-year retrospective study, carried out in Tunisia, showed that rheumatic valve disease was noted in 45.2% of native valve IE. In the study, no instance of IE was related to IV medication use, and staphylococci were the most prevalent microorganisms (17.9%), and congestive heart failure was the most common complication, occurring in 45.2% [24].

A local study in Saudi Arabia stated that native valves were more frequently affected by IE than prosthetic valves. In particular, the mitral valve was the most commonly affected valve. On the other hand, 31.4% of cases had undergone surgical management, with an in-hospital mortality rate of 29.4% [25].

There are not enough studies investigating IE in Saudi Arabia with good, representative sample sizes, and over long periods of time. Our aim is to describe the epidemiological and clinical characteristics and outcomes of adult IE patients.

## Materials and methods

This is a retrospective, descriptive record review study that was approved by the Unit of Biomedical Ethics Research Committee of King Abdulaziz University (KAU). Our aim was to describe the epidemiological and clinical characteristics and outcomes of adult IE patients who were admitted to King Abdulaziz University Hospital (KAUH) in Jeddah, Saudi Arabia, in the period from 2003 until 2019. KAUH is one of the biggest centers in the western region of Saudi Arabia, and it has more than 1000 beds.

We included all adult patients (16 years and above) who were admitted with a definitive diagnosis of IE, according to the Duke Criteria, between 2003 and 2019. Patient data were collected from both paper and electronic medical records. Demographic data (age, gender, nationality, and marital status), type of diagnosis, causative agent, biochemical results, echocardiogram findings, the presence of risk factors, patient comorbidities, and type of management were all documented in our datasheet. The data collection process was confidential, saved and carried out in a secured computer, and all data regarding this study will be removed once completing the study.

Statistical analysis was performed using IBM SPSS Statistics (version 21, IBM Corp., Armonk, NY), and patient confidentiality was ensured. Frequencies, means, standard deviations, and others were calculated to describe the results.

## Results

Out of 105 patients diagnosed with IE, 60 patients met our inclusion criteria, therefore, we excluded 45 patients for the following reasons: 35 were pediatric patients, younger than 16 years old, and 10 either did not have enough data or were possible cases according to the Duke's criteria.

Of the 60 patients, 33 were males (55%) and 27 were females (45%). The mean patient age was 48.71 ± 18.3 years, and the Saudi nationality accounted for 18 patients (30%), while 42 were non-Saudi (70%) (Table 1).

**Table 1:** Demographic data

Feature	No. (%)
Demographic data	
Male	33 (55)
Female	27 (45)
Saudi	18 (30)
Non-Saudi	42 (70)

According to the type of affected valve, 52 of the total patients (86.7%) had native valve involvement, while in 8 (13.3%) prosthetic valves were affected.

The most common comorbidity the patients had was hypertension (HTN), which accounted for 23 patients (38.3%), followed by end-stage renal disease (ESRD) in 20 (33.3%), and DM in 17 (28.3%). Also, 13.3% of the patients were known to have RHD, and they were not only with prosthetic heart valves but also native valves.

Fifty-five of the total IE patients had blood cultures taken, 42 of which were positive (70%), and the most frequent organisms isolated were *S. aureus*, 15 patients (25%), followed by *S. viridans*, 8 (13.3%), and coagulase-negative *S. aureus*, 4 (6.7%) (Table 2).

**Table 2:** Patients' characteristics This table demonstrates patients' characteristics including, predisposing factors, causative organism, type of management, antibiotics used, blood culture result, echocardiography findings, and complications. AKI, acute kidney injury; ESRD, end-stage renal disease; IE, infective endocarditis; MVR, mitral valve regurgitation; AVR, aortic valve regurgitation; TVR, tricuspid valve regurgitation; RHD, rheumatic heart disease.

Feature	No. (%)
Risk factors	
Previous IE	9 (15)
Previous cardiac surgery	11 (18.3)
Prosthetic heart valve	10 (16.7)
Cardiac implantable device	3 (5)
RHD	8 (13.3)
ESRD	20 (33.3)
Previous dental procedure	3 (5)
Previous event of bacteremia or infection	30 (50)
Organisms	
*S. aureus*	15 (25)
*S. viridans*	8 (13.3)
Coagulase-negative *S. aureus*	4 (6.7)
Management	
Medical	58 (96.7)
Surgical	2 (3.3)
Antibiotics	
Vancomycin	15 (25)
Ampicillin	7 (11.7)
Ceftriaxone	7 (11.7)
Chemotherapy	1 (1.7)
Blood culture	
Positive	42 (70)
Negative	13 (21.7)
Echocardiography findings	
Vegetations	53 (88.3)
MVR	17 (28.3)
AVR	14 (15)
TVR	3 (5)
Complications	
Sepsis	16 (26.7)
Stroke	4 (6.7)
Pulmonary emboli	4 (6.7)
AKI	4 (6.7)
Brain emboli	3 (5)
Multi-organ failure	2 (3.3)
Septic emboli	2 (3.3)
Heart failure	2 (3.3)
Aortic root abscess	1 (1.7)
Septic shock	1 (1.7)

Twenty-one of the total IE patients had C-reactive protein (CRP) laboratory tests done, and 18 of them showed high levels (30%). Twenty-one of the patients had erythrocyte sedimentation rates (ESR) evaluated, and ESR was high in 19 patients (31.7%). All the IE patients had white blood cell (WBC) counts tested: 35 showed leukocytosis (58.3%), 22 had normal counts (36.7%), and 3 had leukocytopenia (5%).

Of the total 60 IE patients, 53 (88.3%) had vegetations. The anatomical locations of vegetations were as follows: mitral valve 28 (46.7%), aortic valve 17 (28.3%), tricuspid valve 7 (11.7%), and pulmonary valve 3 (5%). Reports of 3 of the 53 patients did not document the locations of vegetations. Only one patient was found to have vegetations on a pacemaker wire. The majority of patients had a solitary vegetation 42 (79.2%). Echocardiogram for 35 patients (58.3%) showed valvular destruction. Nine patients (15%) presented with severe aortic valve regurgitation, and 8 (13.3%) presented with severe mitral regurgitation. Ejection fraction was found within the normal range in 36 (69.2%) and was reduced in 8 (15.4%). Also, ejection fraction at the first follow-up was within the normal range in 12 patients (20%); however, for 46 patients, there were missing reports. On the other hand, 29 (48.3%) presented with complications, and sepsis was found in 16 (26.7%), followed by stroke, pulmonary emboli, and AKI by (6.7%) for each. The mortality rate was 22 (36.7%).

## Discussion

In this review of cases over 17 years, we found 60 cases that met our inclusion criteria, with a mean age of 48.71 years, and a male to female ratio of 1.2:1. Nashmi and Memish had conducted a local study in Riyadh and found a total incidence of 47 patients diagnosed with IE over 10 years. Their mean age was 38 years, with a 1.6:1 male to female ratio [13].

Regarding IE risk factors, we found that 33.3% of the patients had ESRD, 18.3% had previous cardiac surgery, 16.7% had prosthetic heart valves, 15% had a history of previous IE, 13.3% had a positive history of RHD, and 5% had previous dental procedures. Simsek-Yavuz et al. had observed that prosthetic valves and RHD were the two most frequent risk factors, with percentages of 43.5% and 33.9%, respectively. Moreover, they stated that chronic renal failure and being on dialysis have been associated with increased mortality rates in IE patients [23].

We discovered that 55 of the total IE patients had blood cultures taken, 42 of which were positive (70%), and the most common organisms isolated were *S. aureus*, 15 (25%), then *S. viridians,* 8 (13.3%), and coagulase-negative *S. aureus*, 4 (6.7%).

These results are compatible with a study that was done in the United States (US) in 2015, which reported that *S. aureus* remains to be the most common cause of IE [20]. This similarity in results can be clarified by the reality that *S. aureus* is the most prevalent cause of all types of IE [26].

On the other hand, there is a previous study, also carried out in the US, from 2018 that found that the most commonly reported organism was *S. viridians* followed by *S. aureus* [27].

The conflicting research results may be owing to the modifications in the guidelines that occurred in 2007 [20]. The modifications in the population of patients with IE influenced the trends in the bacterial identity of IE [28].

Several types of research confirmed that the most common site for vegetations was the mitral valve followed by the aortic valve [22,25]. Besides that expected result for the location of vegetations, we also found that severe regurgitation comes up slightly more often with aortic than with mitral valve involvement. Alongside the severity of valve destruction, we established that the ejection fraction remains roughly within the normal range, as it does at the next follow-up. On the other hand, a study done in Yemen documented ejection fractions below 40%. Furthermore, they found that the most common complication was heart failure, while in our study, sepsis was the highest occurring complication [29]. It is wise to consider that a variety of our patients seemed to be immunocompromised due to DM and other chronic diseases or medications they were receiving, such as corticosteroids. Unfortunately, mortality was still high in our study with a percentage of 36.7%. A systematic review and a meta-analysis published in 2017 showed the mortality rate in the short term to be 20%, while in the long term, 37% [30]. Additionally, a study done in Dhahran in 2009 reported a mortality rate of 29.4% [25]; and research performed in Turkey in 2015 reported a mortality rate of 27.8% [23].

Regretfully, the explanation behind our high mortality rate could include the high extent of comorbidities our patients had, the high mortality rate of the most frequent causative organism (*S. aureus*), lack of prompt surgical intervention, and surgical facilities (when they are indicated) because our cardiac surgery center was established in 2006 while our review included patients from as far back as 2002. In this sense, our paper could have some degree of referral bias. Also, IE is still quite uncommon among citizens and residents in Saudi Arabia.

Sepsis was the trending complication in our data, followed by stroke and pulmonary emboli. On the other hand, Heiro et al. demonstrated that neurological complications were the most frequent, proceeded by peripheral emboli [6].

## Conclusions

There was a small number of definitive IE cases over a 17-year span in our institution. Surprisingly, a higher mortality rate was found in our patients and a lower surgical intervention rate in comparison with the literature. Though we acknowledge the limitations of a retrospective chart review study, we can speculate that the high mortality rate may be in part related to higher numbers of virulent organisms, delayed presentation, and lack of prompt surgical intervention.
